# Soy isoflavone consumption and colorectal cancer risk: a systematic review and meta-analysis

**DOI:** 10.1038/srep25939

**Published:** 2016-05-12

**Authors:** Yi Yu, Xiaoli Jing, Hui Li, Xiang Zhao, Dongping Wang

**Affiliations:** 1 Emergency Department, The First Affiliated Hospital of Sun Yat-sen University, Guangzhou, Guangdong Province, People’s Republic of China; 2 Organ Transplant Center, The First Affiliated Hospital of Sun Yat-sen University, Guangzhou, Guangdong Province, People’s Republic of China

## Abstract

Colorectal cancer (CRC) is one of the most predominant solid carcinomas in Western countries. However, there is conflicting information on the effects of soy isoflavone on CRC risk. Therefore, we performed a meta-analysis to assess the association between soy isoflavone consumption and CRC risk in humans using PubMed, Embase, Web of Science, and Cochrane Library databases. A total of 17 epidemiologic studies, which consisted of thirteen case-control and four prospective cohort studies, met the inclusion criteria. Our research findings revealed that soy isoflavone consumption reduced CRC risk (relative risk, RR: 0.78, 95% CI: 0.72–0.85; *I*^*2*^ = 34.1%, *P* = 0.024). Based on subgroup analyses, a significant protective effect was observed with soy foods/products (RR: 0.79; 95% CI: 0.69–0.89), in Asian populations (RR: 0.79; 95% CI: 0.72–0.87), and in case-control studies (RR: 0.76; 95% CI: 0.68–0.84). Therefore, soy isoflavone consumption was significantly associated with a reduced risk of CRC risk, particularly with soy foods/products, in Asian populations, and in case-control studies. However, due to the limited number of studies, other factors may affect this association.

Colorectal cancer (CRC) is the third most prevalent cancer worldwide and one of the most common solid carcinomas in Western countries^1^. Therefore, primary CRC prevention efforts should be explored. Based on recent estimates, the CRC incidence rate is higher in developed nations than in developing countries^2^. Lifestyle habits and diet may play key roles in the etiology of CRC^3^,^4^. Soy isoflavones, which are phytoestrogens, have a protective effect against cancer formation and susceptibility to radiotherapy; other anti-cancer phytochemicals in soy beans include phenolic acids, plant sterol, and protease inhibitors^5^,^6^,^7^,^8^,^9^,^10^.

The association between soy isoflavone consumption and CRC risk has been evaluated^11^,^12^. A meta-analysis of four cohort studies and seven case-control studies failed to detect any association between soy consumption and risk of CRC, colon cancer, or rectal cancer^12^. Due to a low statistical power and small sample size of each individual study, the results were not consistent with the findings of several epidemiological studies^13^,^14^,^15^,^16^. Therefore, the objective of this meta-analysis was to assess the association between soy isoflavone consumption and CRC risk. Our meta-analysis included case-control and cohort studies^13^,^14^,^15^,^16^,^17^,^18^,^19^,^20^,^21^,^22^,^23^,^24^,^25^,^26^,^27^,^28^,^29^ and subgroup analyses by geographic area, study type, anatomical subsite, gender, and soy food type.

## Methods

### Search strategy

We performed a literature search of relevant studies published through November, 2015 using PubMed (http://www.ncbi.nlm.nih.gov/pubmed/), Embase (http://www.embase.com/), Web of Science (http://wokinfo.com/), and Cochrane Library (http://www.thecochranelibrary.com/). The search strategy included terms for outcome (colorectal neoplasm, colorectal cancer, colon cancer, and rectal cancer) and exposure (soy, soy foods/products, isoflavones, soybeans, flavonoid, tofu, soy protein, miso, genistein, phytoestrogen, and natto). We designed, implemented, and reported our meta-analysis based on epidemiological study guidelines^30^. In addition, we reviewed the reference lists from all relevant articles to identify additional studies. A search for unpublished literature was not performed.

### Study selection

The study inclusion criteria were the following, (i) studies written in English with case-control or cohort design; (ii) original human clinical trials that evaluated the association between soy isoflavone consumption and CRC risk; and (iii) use of risk point estimates, e.g., odd ratio (OR), relative risk (RR), or hazard ratio (HR) estimates with 95% confidence intervals (CIs).

### Data extraction

The extracted data were the first author’s name, year of publication, cancer type, population and country, total number of cases, dietary assessment method, estimates of soy isoflavone intake, and RRs or ORs with 95% CIs. Five publications reported separate RRs for soy foods and soy isoflavones, five publications reported separate RRs for male and female participants, and two publications reported separate RRs for colon and rectal cancers. In these cases, RRs were extracted individually.

### Statistical analysis

We assessed the association between soy isoflavone consumption and CRC risk using the reported RRs. Soy isoflavones were defined as soy foods, soy products, isoflavones, tofu, soy milk, miso, natto, genistein, daidzein, and flavonols. When adjusted and crude RRs were provided, the most adjusted RRs were extracted.

We used HR and OR to evaluate CRC risk. HR and OR were considered to be approximations to RR, because CRC is a rare outcome in humans. Pooled RRs and 95% CIs were estimated on the basis of the most adjusted RRs or ORs for the highest versus lowest soy isoflavone intake.

We used *I*^*2*^ and *Q* statistics to assess possible homogeneity of RRs across studies, which is a quantitative measure of inconsistency among studies^31^. Pooled ORs and 95% CIs were calculated using a random effects model^32^. To estimate cancer site-specific and ethnicity-specific effects, subgroup analyses were performed by geographic area, study type, anatomical subsite, gender, and soy isoflavone type. Additionally, a sensitivity analysis was conducted to investigate the effect of a single study on the overall risk estimate. This allowed us to estimate whether the results could have been significantly affected by a single study.

Data analyses were performed with STATA version 13.0. Statistical significance was set at *P* ≤ 0.05. Egger’s and Begger’s regression models were used to evaluate potential publication bias^31^. All reported *P* values were from two-sided statistical tests.

## Results

**Figure 1:**
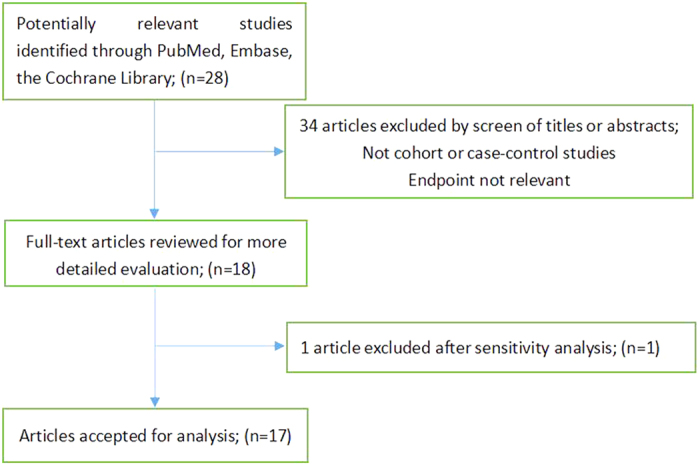
Search strategy and selection of studies.

**Figure 2:**
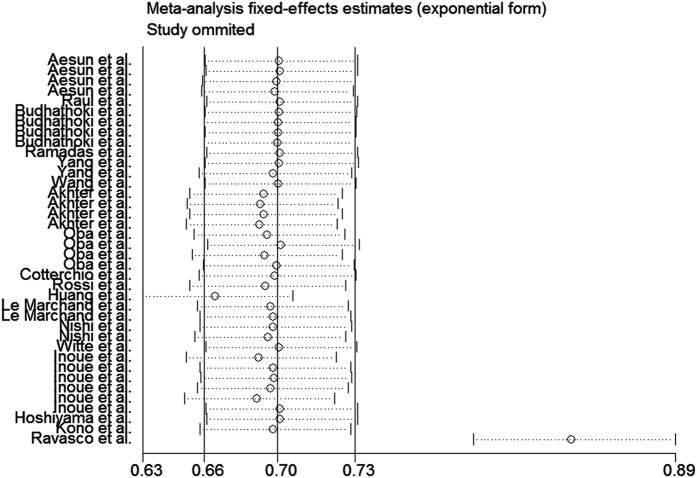
Sensitivity analysis of soy isoflavone consumption and risk of colorectal cancer excluding the study of Ravasco *et al*.^33^.

The study selection process is graphically described in Fig. 1. Twenty studies met our inclusion criteria. Two studies were subsequently excluded, because one was an ecological study and the other study failed to report RR or 95% CI. After conducting a sensitivity analysis, we excluded the Ravasco *et al*. study^33^ (Fig. 2). Finally, 17 studies^13^,^14^,^15^,^16^,^17^,^18^,^19^,^20^,^21^,^22^,^23^,^24^,^25^,^26^,^27^,^28^,^29^ were included in the meta-analysis (Table 1). The most predominant dietary assessment method used in these studies was the food frequency questionnaire (FFQ).

Thirteen studies assessed the association between soy product consumption and CRC risk, while nine studies evaluated the association between isoflavone consumption and CRC risk. Among them, six studies separately presented findings for men and women, and two studies separately reported results for risk of rectal and colon cancers. Twelve studies were conducted in Asia and five in non-Asia countries (Table 2). Data from both men and women were individually extracted. Different soy food types were evaluated in these studies; some studies assessed more than one type of soy food. Therefore, we used the risk estimate that was the most representative of overall soy consumption and the soy food item that was the most commonly consumed. In descending order, the most common soy food or products were tofu (bean curd), soy beans, soy milk, and miso soup (soy paste soup).

**Figure 3:**
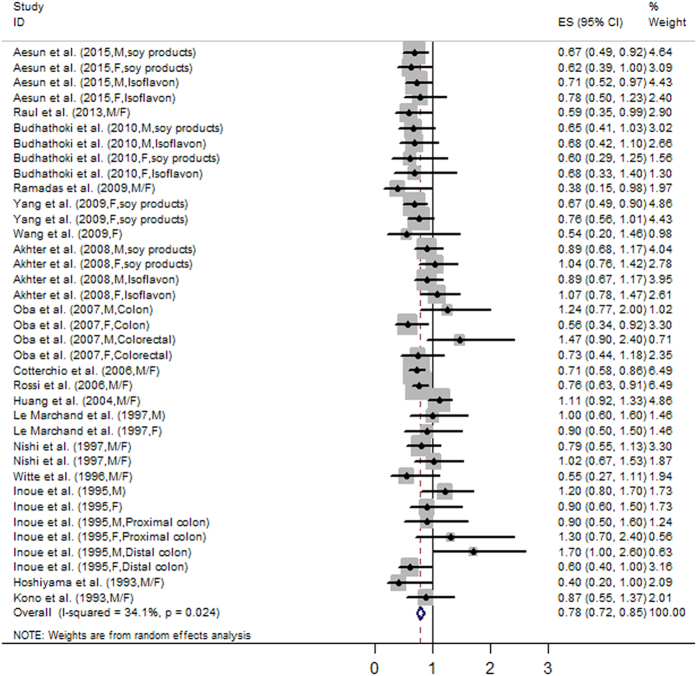
Forest plot of studies evaluating the association between soy isoflavone consumption and risk of colorectal cancer.

**Figure 4:**
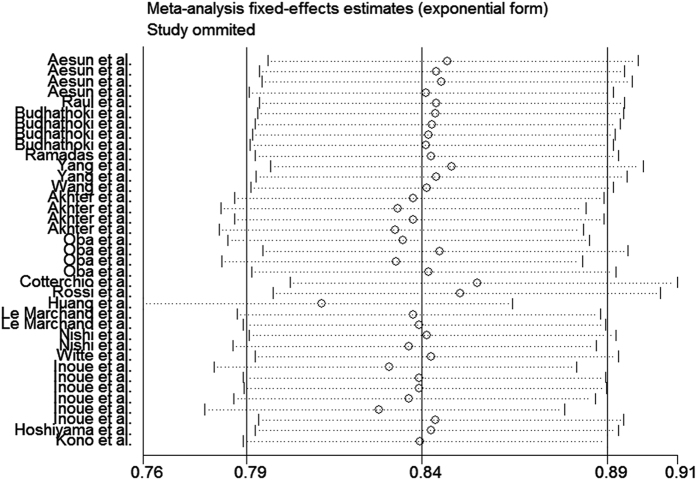
Sensitivity analysis of soy isoflavone consumption and risk of colorectal cancer.

**Figure 5:**
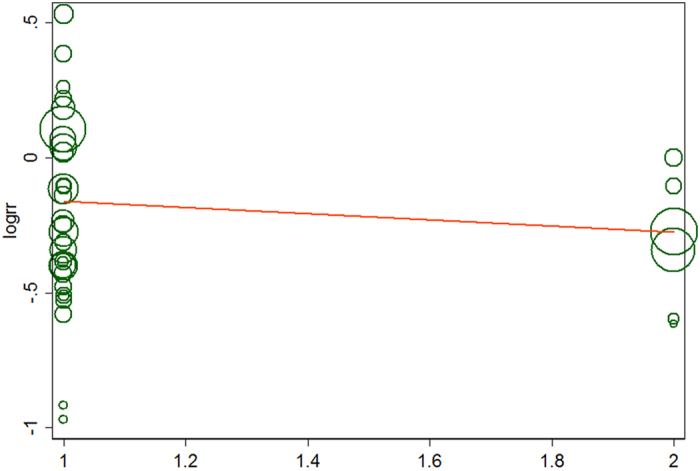
Meta regulation of isoflavone consumption and risk of colorectal cancer.

**Figure 6:**
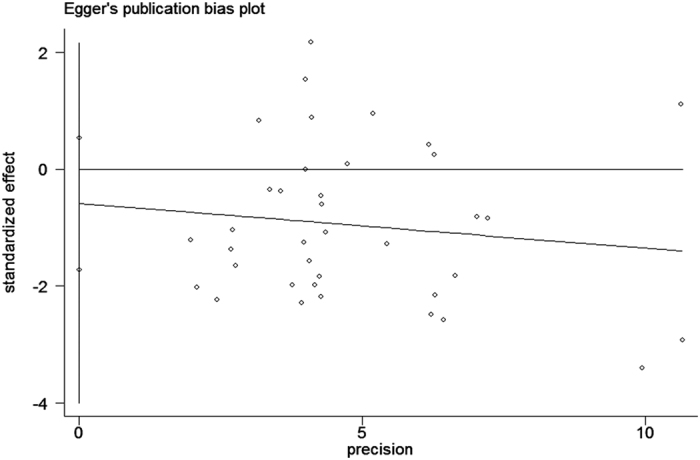
Egger’s funnel plot assessing publication bias among the studies.

**Figure 7:**
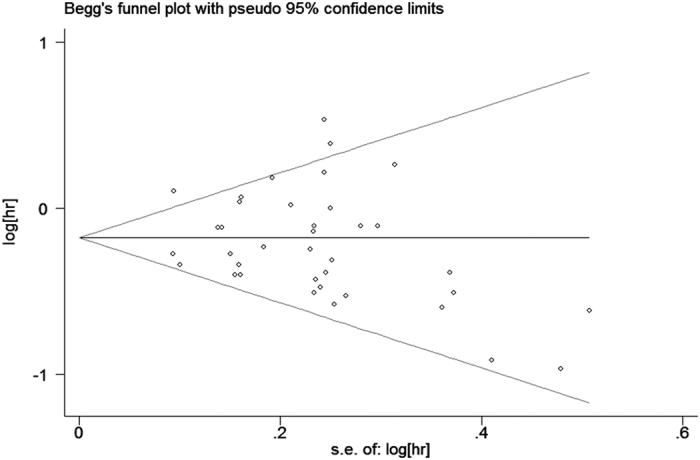
Begger’s funnel plot assessing publication bias among the studies.

The analysis of the 17 studies yielded a combined risk estimate of 0.77 (95% CI, 0.72–0.82; *P* = 0.024) with a heterogeneity value (*I*^*2*^) of 34.1% (Fig. 3). However, the results from the 17 studies were inconsistent. Nine studies reported that soy isoflavone intake was associated with a significant reduction in CRC risk, whereas other studies reported no association. Six studies reported that soy isoflavone intake was associated with a significant reduction in CRC risk in both men and women, three studies reported a significant reduction in CRC risk only in women, and other studies reported no association in women or men. We conducted a sensitivity analysis (Fig. 4) and meta regulation test (Fig. 5). The sensitivity analysis revealed that the publication dates were similar. The geographical area was associated with ~44.3% heterogeneity reduction across the studies. No publication bias was detected (Figs 6 and 7) based on Egger’s and Begger’s regression models^32^.

Because there were differences in study types (cohort or case-control), study populations (Asian or non-Asian), anatomical subsite (colorectal, colon, or rectum), gender (female versus male), and soy isoflavone type (soy foods/products or soy isoflavones) among the studies, we further conducted subgroup analyses to determine the effect of these factors in our analyses (Table 2). We obtained a statistically significant protective effect of soy foods/products (RR: 0.79; 95% CI: 0.72–0.84), in Asian populations (RR: 0.79; 95% CI: 0.73–0.85), and with case-control studies (RR: 0.76; 95% CI: 0.70–0.81).

## Discussion

We analyzed 17 epidemiological studies that assessed the association between soy isoflavone consumption and CRC risk in humans. The findings revealed that the consumption of soy isoflavones was associated with a 23% reduction in CRC risk. CRC is caused by environmental (e.g., diet and lifestyle) and genetic factors^34^. When stratified by geographical area, a significant protective effect of soy isoflavone consumption was observed in Asian populations, which are likely to be attributed to their lifestyle habits and overall health. Ecological and immigration studies have shown that differences in CRC risk among populations are largely attributed to environmental factors, such as eating habits. Asian populations have higher intakes of soy isoflavones than Western populations^35^. The consumption of Western diets, which are high in fat and calories, is associated with an increased incidence in CRC. Dietary fat increases the secretion of bile acids, which directly damage the intestinal mucosa, stimulate epithelial hyperplasia, and increase CRC risk^36^. On the other hand, the frequency of physical activity is lower in Asian populations than in American or European populations. Regular physical activity is a protective factor against CRC, because it reduces random motions of the intestine and stimulates bowel movements. Additionally, physical activity promotes the secretion of prostaglandins, which stimulate peristalsis and cleansing and reduce the contact time between the intestinal mucosa and carcinogens^37^,^38^. When stratified by study design, a significant protective effect of soy isoflavone intake was observed with case-control studies, which could be attributed to higher recall rates and greater selection bias in these types of studies. When stratified by soy foods/products and soy isoflavones, a significant protective effect was observed with soy foods/products, probably due to a limited number of studies focused on soy isoflavones.

Epidemiological and animal studies have found that the consumption of dietary soy decreases the incidence of certain tumors, including those of the colon and rectum^39^,^40^,^41^,^42^,^43^. The three main soy isoflavone aglycones are genistein, daidzein, and glycitein^43^. The mechanism by which soy protects against the development of CRC remains unclear. It has been reported that in CRC, there is a reduced expression of estrogen receptor-*β* (ER-*β*) expression^44^. Dietary isoflavones increase ER-*β* expression, but reduce ER-*α* expression in the colon of female rats^45^. In CRC patients, ER gene expression is either diminished or absent^46^.

Our meta-analysis had some limitations. First, only studies written in English were included. Second, most studies used FFQs as the main dietary assessment method. Recall bias may have affected the results. Additionally, it was challenging to predict the effect of misclassification of case-control studies on the results. Third, certain confounding factors were not adjusted in the evaluated studies, e.g., family history of CRC, smoking, and alcohol consumption, which are important risk factors of CRC^47^,^48^,^49^. Fourth, we failed to evaluate a dose-response relationship between soy isoflavone consumption and CRC risk.

There was heterogeneity across the studies in terms of soy isoflavone consumption, which is not surprising considering the differences in the study designs, soy types, and gender. Additionally, differences in geographic area may have contributed to the heterogeneity results; most of the studies were conducted in Asia, where the consumption of soy is high. Moreover, while some studies were adjusted for age, gender, and family history of CRC in the calculation of risk estimates, not all parameters were considered. The measurement units varied among the studies. Sensitivity analysis was performed by sequentially omitting one single study to assess the effect of each study on the overall results (Fig. 2). The Egger’s funnel plot revealed a *P* value > 0.05; the shape of the Begger’s funnel plot seemed symmetrical. There was no significant evidence for publication bias in our meta-analysis (*P* > 0.05).

In summary, our meta-analysis provided an updated and comprehensive evaluation of the association between soy isoflavone consumption and CRC risk, with an RR value of 0.77 (95% CI: 0.72–0.82, *P* = 0.024) and an *I*^*2*^ value of 34.1%. A statistically significant protective effect was observed with soy foods/products (RR: 0.79; 95% CI: 0.72–0.84), in Asian populations (RR: 0.79; 95% CI: 0.73–0.85), and with case-control study designs (RR: 0.76; 95% CI: 0.70–0.81). Soy isoflavones play an important protective role in the pathogenesis of CRC, by a mechanism that remains to be elucidated. More cohort and intervention studies are required.

## Additional Information

**How to cite this article**: Yu, Y. *et al*. Soy isoflavone consumption and colorectal cancer risk: a systematic review and meta-analysis. *Sci. Rep.* **6**, 25939; doi: 10.1038/srep25939 (2016).

## Figures and Tables

**Table 1:** Characteristics of the studies included in the meta-analysis.

Study	Year	Cancer type	Population and country	No. of cases	Dietary assessment	Exposure	Consumption comparison	Adjustment RR (95% CI)	Adjustment
Aesun *et al*.	2015	CRC	2,669 Korea	901	SQFFQ	Soy products	Highest versus lowest	Colorectal 0.67 (0.49–0.92), M 0.62 (0.39–1.00), F	Age, education, alcohol consumption, and regular physical activity
						Isoflavones	Highest versus lowest	Colorectal 0.71 (0.52–0.97), M 0.78 (0.50–1.23), F	
Raul *et al*.	2013	CRC	825 Spain	424	FFQ	Isoflavones	Highest versus lowest	Colorectal 0.59 (0.35–0.99)	Sex, age, BMI, energy intake, alcohol and fiber intake
Budhathoki *et al*.	2010	CRC	1,631 Japan	816	FFQ	Soy foods	37.2 (23.0–54.9) (mg/d) versus 35.5 (22.3–52.3) (mg/d)	Colorectal 0.65 (0.41–1.03), M 0.60 (0.29–1.25), F	Age, resident area, parental colorectal cancer, smoking, alcohol consumption, BMI, job, leisure-time physical activity, and energy-adjusted intakes of calcium and n-3 polyunsaturated fatty acids
						Isoflavones		0.68 (0.42–1.10), M 0.68 (0.33–1.40), F	
Ramadas *et al*.	2009	CRC	108 Malaysia	59	FFQ	Soy products	<3 times/week versus ≥3 times/week	Colorectal 0.38 (0.15–0.98)	Age, ethnicity, gender, physical activity, height, BMI, waist circumference, energy intake, current alcohol consumption and smoking habits
Yang *et al*.	2009	CRC	68,412 women China	321	FFQ	Soy foods	≤12.8 versus >21.0 g/d	Colorectal 0.67 (0.49–0.90), F	Birth calendar year, education, BMI, household income, physical activity, colorectal cancer in first-degree relatives, menopausal status, and dietary intakes of total calories, red meat, total fruit and vegetables, non-soy fiber, non-soy calcium, and non-soy folic acid
						Isoflavones	≤15.1 versus >48.9 mg/d	0.76 (0.56–1.01), F	
Wang *et al*.	2009	CRC	38,408 women US	301	SFFQ	Soy foods (Tofu)	<1 time/month versus ≥1 time/week	Colorectal 0.54 (0.20–1.46), F	Age, race, total energy intake, and randomized treatment assignment, BMI, smoking, alcohol consumption, physical activity, postmenopausal status, hormone replacement therapy use, multivitamin intake, family history of cancer in a parent or sibling, and intake of fruit and vegetables, fiber, folate, and saturated fat
Akhter *et al*.	2008	CRC	38,408women Japan	886	FFQ	Soy foods	≤35.4 versus >169.9 g/d, M ≤35.6 versus >170.3 g/d, F	Colorectal 0.89 (0.68–1.17), M 1.04 (0.76–1.42), F	Age, public health center, area, history of diabetes mellitus, BMI, leisure time physical activity, cigarette smoking, alcohol drinking, energy-intake, menopausal status, use of female hormones
						Isoflavones (Genistein)	≤9.1 versus >50.4 mg/d, M	0.89 (0.67–1.17), M 1.07 (0.78–1.47), F	
Oba *et al*.	2007	Colon cancer	30,221 Japan	213	FFQ	Soy products	≤49.22 versus >141.09 g/d, M	Colon 1.24 (0.77–2.00), M 0.56 (0.34–0.92), F	Age, physical activity, cigarette smoking status, height, BMI, alcohol and coffee consumption, hormone replacement therapy (for women)
						Isoflavones	22.45 versus 59.58 mg/d, M	1.47 (0.90–2.40), M 0.73 (0.44–1.18), F	
Cotterchio *et al*.	2006	CRC	1,890 Canada	1,095	FFQ	Isoflavones	0 versus >1.097 mg/d	0.71 (0.58–0.86)	Age, sex, and total energy intake
Rossi *et al*.	2006	CRC	4,154 Italy	1,953	FFQ	Isoflavones	≤14.4 versus >33.9 μg/d	0.76 (0.63–0.91)	Age, sex, study center, family history of colorectal cancer, education, alcohol consumption, BMI, occupational physical activity, and energy intake
Huang *et al*.	2004	CRC	50,706 Japan	1,352	FFQ	Bean curd	<3 versus ≥ 3 times/week	Colorectal 1.11 (0.92–1.33)	Age and sex
Nishi *et al*.	1997	Colon Cancer Rectal cancer	660 Japan	330	FFQ	Soy products (Tofu)	<3 versus ≥ 3 times/week	Colon 0.79 (0.55–1.13) Rectum 1.02 (0.67–1.53)	Age, sex and registered residence
Le Marchan *et al*.	1997	CRC	1,192 US	1,192	FFQ	Tofu	0 versus ≥25 g/d	Colorectal 1.0 (0.6–1.6), M 0.9 (0.5–1.5), F	Nutrient intakes for calories, age, family history of colorectal cancer, alcoholic drink, cigarette smoking, Quetelet index previous five year, total calories, egg intake, lifetime recreational activity (in hours), and calcium intake
Witte *et al*.	1996	CRC	488 US	488	FFQ	Tofu or Soy beans	None versus ≥1 serving/week	Colorectal 0.55 (0.27–1.11)	Race; body mass index); vigorous leisure time activity; smoking; dietary fiber, folate, beta-carotene, and vitamin C
Inoue *et al*.	1995	Rectal Cancer	31,782 Japan	432	FFQ	Bean curd	≤3 versus >3 times/week	Proximal colon 0.9 (0.5–1.6), M 1.3 (0.7–2.4), F Distal colon 1.7 (1.0–2.6), M 0.6 (0.4–1.0), F Rectum 1.2 (0.8–1.7), M 0.9 (0.6–1.5), F	Age
Hoshiyama *et al*.	1993	Colon Cancer	653 Japan	181	FFQ	Soy bean	≤4 versus ≥8 times/week	Colon 0.6 (0.3–1.3) Rectum 0.4 (0.2–1.0)	Sex, age for colon cancer, selected food items; sex and age for rectal cancer
Kono *et al*.	1993	Colon Cancer	1,557 Japan	187	FFQ	Soy paste soup	<1 versus ≥2 bowls/d	Colon 0.87 (0.55–1.37)	Smoking, alcohol consumption, rank and BMI

BMI: body mass index, CI: confidence interval, FFQ: food frequency questionnaire, RR: relative risk, SQFFQ: semi-quantitative food frequency questionnaire; F: female, M: male, Null: not provided.

**Table 2:** Stratified analysis of colorectal cancer in relation to soy isoflavone consumption according to study characteristics.

Group	No. of studies	RR (95% CI)	*P*_heterogeneity_	*I*^*2*^ (%)
Soy types and colorectal cancer				
Soy foods/products	14	0.79 (0.69–0.89)	0.006	46.2%
Isoflavones	8	0.76 (0.69–0.83)	0.559	0
Gender				
Male	6	0.86 (0.73–0.99)	0.085	38.4%
Female	8	0.74 (0.66–0.83)	0.493	0
anatomical subsite				
Colorectal	12	0.77 (0.70–0.84)	0.092	28.6%
Colon	4	0.85 (0.64–1.05)	0.082	44.6%
Rectum	3	0.87 (0.52–1.22)	0.05	61.7%
Study types				
Cohort	4	0.83 (0.71–0.95)	0.118	35.1%
Case-control	13	0.76 (0.68–0.84)	0.045	34.4%
Geographic area				
Asia	12	0.79 (0.72–0.87)	0.01	41.1%
Non-Asia	5	0.74 (0.64–0.83)	0.722	0

No, number; RR, relative risk; CI, confidence interval.
